# How the Indians Climb Trees

**Published:** 1874-12

**Authors:** 


How the Indians Climb Trees.—In South America even the weakest women may not uncommonly be seen plucking the fruit at the tree tops. If the bark is so smooth and slippery that they cannot go up by climbing, they use other means. They make a hoop of wild vines, and putting their feet inside, they use it as a support in climbing. The negro of the West coast of Africa makes a large hoop round the tree and gets inside of it, and jerks it up the trunk with his hands, a little at a time, drawing his legs after it. The Thaitlan boys tie their feet together, four or five inches apart, with a piece of palm bark, and with the aid of this fetter go up the cocoa palms to gather nuts. The native women of Australia climb the gum trees after opossums; where the bark is rough they chop holes with a hatchet, then one throws about the tree a rope twice as long as will go around it, puts her hatchet on her cropped head, and, placing her feet against the tree and grasping the rope with her hands, she hitches it up by jerks, and pulls herself up the enormous trunk almost as fast as a man will climb a ladder.

